# Exploring the Social and Emotional Representations Used by the Elderly to Deal With the COVID-19 Pandemic

**DOI:** 10.3389/fpsyg.2020.586560

**Published:** 2021-01-27

**Authors:** Amaia Eiguren, Nahia Idoiaga, Naiara Berasategi, Maitane Picaza

**Affiliations:** ^1^Department of Didactics and School Organization, University of the Basque Country - UPV/EHU,, Bilbao, Spain; ^2^Department of Evolutionary and Educational Psychology, University of the Basque Country - UPV/EHU, Bilbao, Spain

**Keywords:** COVID-19, elderly, emotions, pandemic, social representations

## Abstract

Spain has become one of the European epicenters of coronavirus (COVID-19), a virus that particularly affects the elderly, since this group accounts for the majority of hospitalized cases and has the highest mortality rates. Therefore, the aim of this research is to understand how elderly people represent and emotionally cope with COVID-19 during the days when the pandemic emerged in Spain. Using a qualitative methodology, a free association exercise elicited by the word “COVID-19” was completed by 115 participants (age range: 60–85 years) from the North of Spain. Lexical analysis was used to analyze the content. The results revealed that the government and the mass media are criticized for failing to communicate a clear message, and for giving out information that is both insufficient and contradictory. However, participants are clear that it is essential to follow the guidelines of the scientists and doctors, which are represented as credible sources. However, when the state of alarm and the lockdown of all citizens was declared, most of the participants represented the risk as being associated with the elderly and the pandemic became something that might also affect their families. Due to these circumstances, negative emotions appear such as fear, nervousness, uncertainty, restlessness, and insecurity. Feelings of solitude and loneliness also emerged, and these are represented as being linked to death. These results indicate the need for governments to manage the current situation with the elderly by placing greater emphasis on social and inclusive policies to help alleviate the possible effects of the pandemic and the lockdown.

## Introduction

In December 2019, the new coronavirus (COVID-19) emerged in Wuhan (China) and became the focus of a pneumonia epidemic of unknown origin (Sahin et al., 2020). Between the months of January and February 2020, this new Emerging Infectious Disease (EID) began to spread outside of China (Liu et al., 2020). Europe – particularly Italy and Spain – became important centers of the pandemic with a notable increase in the number of infections and deaths, particularly among the elderly (Linde, 2020).

Due to the increase in infections, on March 14th the Spanish Government declared a state of emergency and ordered the entire population to remain in lockdown (Aragó, 2020; National Epidemiological Surveillance Network, 2020). Since the beginning of the epidemic, the Spanish Ministry of Health, Consumers and Social Welfare (2020) has placed special emphasis on recommendations for elderly people, regarding them as a high-risk group, following the indications of the mortality data in China (BBC, 2020; Ramos, 2020). From the executive branch, the Spanish prime minister, Pedro Sánchez, advised at the press conference following the extraordinary Council of Ministers in early March that the population, particularly the elderly or those with chronic diseases, should remain confined to their homes or retirement homes as a preventive measure (Berreiro and Rodriguez, 2020; Presidency of the Government of Spain, 2020; Spanish Society of Geriatric Medicine, 2020).

In the Basque Autonomous Community (in Northern Spain) where the present research was conducted, cases of COVID-19 only began to be visible at the beginning of March, but the number of infections increased rapidly. According to a report by the Basque Government’s Health Department (2020) published on 14th March, there were 593 positive cases and 3 days later, on 17th March, the total number of positive cases had risen to 973. On 18th March, 50.7% of people affected by the COVID-19 were over-aged 60 years with the death rate standing at 49.4% for this group of people. Specifically, 47 of the 50 people who died were aged over 60. In the first 3 months of the pandemic (until June 14, 2020) 20,415 cases were diagnosed in the Basque Country and there were 1,592 deaths from COVID-19, of which 73.2% were 60 years or older (Basque Government, 2020). Thus, on a global scale, the elderly currently represents one of the largest vulnerable groups in this health crisis (Hernández, 2020).

The world in which we live has changed overnight as a result of these unprecedented events, and, as demonstrated in previous EIDs, this has a profound impact on society (Washer, 2010). In order to tackle this challenge, the Social Representations Theory (SRT) created by psychologist Serge Moscovici (1961, 1984, 1988), offers us a perspective for understanding not only people’s everyday thinking but also their social strategies for dealing with this new risk. After all, the objective of this academic perspective on social representations is to understand how people internalize and explain new events or risks that change the world as they have known it up until now (as is the case with COVID-19).

Within this representation, it is key to understand that EIDs in general, of which COVID-19 is a clear example, break down the barriers between the global and the local, whilst being, to a certain extent, simultaneously local and global phenomena (Robertson, 1992). In fact, the risks posed by EIDs have no frontiers and thus the risk of contagion is a matter that transcends the boundaries of space and time (Beck, 2009). The globalization of risk in itself is what makes society feel, understand and assimilate the risk and this is an indispensable premise of their social representation and a basis for understanding how epidemics become embedded in our everyday thinking (Idoiaga et al., 2017a).

With regard to social representations of specific health epidemics, extensive research (Joffe and Haarhoff, 2002; Joffe and Bettega, 2003; Joffe and Lee, 2004; Washer, 2006; Idoiaga et al., 2017a,b) has shown that in this society of risk in which we live, risks easily reach us and thus change from being something abstract or distant to being something that is very real and that has a direct impact on us as individuals. In these cases, without the possibility of attributing the risk to “the others,” EIDs are represented in terms of local heroes, victims, and villains (Wagner-Egger et al., 2011). These characteristics could help in the construction of a symbolic representation that enables lay people to make sense (Wagner et al., 2002) of conflicting and discordant pieces of information spread by the media and mentioned in everyday conversations (Wagner-Egger et al., 2011).

First, the heroes of EIDs are the scientific and medical experts (e.g., doctors, scientists), who are mainly perceived as credible and trustworthy sources. Second, the villains of health crises are the media, accused of using fear for their own gain and, even worse, being perceived as the puppets of evil powers at the highest level (Idoiaga et al., 2017b). In addition, governments are also regarded as villains due to acts of corruption and concealment of the problem that facilitated the spread of the disease (Washer, 2006). Some authors have even pointed out that the decisions made by the institutions are represented as being guided by political or economic interests as opposed to health concerns (Smith, 2006). However, other studies have concluded that there are still ambivalent emotions towards the authorities since the health and political authorities tend to be viewed in a positive light at the start of a health crisis, after which there is an eventual tendency for the public to perceive them as ineffective (Wagner-Egger et al., 2011). Finally, it is the infected people that are usually represented as the victims, particularly those who, either because they belong to a risk group or live in countries with a poor health system, are defenseless in the face of the epidemic (Idoiaga et al., 2017a).

However, we should not think that the representation of risk is homogeneous throughout society. The SRT also states that it is precisely in moments of crisis when shared and socially constructed identity ideas emerge spontaneously among different groups (Wagner and Hayes, 2005; Washer, 2006) and that group identity is essential for constructing the representation of risk (Joffe, 2003). Social representations are important for this relationship because these are a way of dealing with a risk to the personal or collective identity, with defense against the threat being one of their main objectives (Moscovici and Duveen, 2000).

One important element of group identity in risk construction processes is the identity of the vulnerability of different social groups. In fact, in representations of health, perceived group invulnerability or vulnerability is of vital importance in protecting oneself (Rossetto et al., 2011), since it influences an individual’s capacity to respond to the health crisis (Delor and Hubert, 2000). From this perspective, age is a key factor in representing the identity of the vulnerability of people in relation to an EID. Aging is often regarded as being synonymous with poor health and deterioration (Coupland and Coupland, 1990), which is likely to have implications for the attribution of risk representation, as well as the perceived tendency to suffer the negative consequences of an epidemic.

Indeed, in this COVID-19 epidemic, it has been stressed from the outset that the elderly constitute the largest global risk group (World Health Organization, 2020). In fact, this is the group that accounts for the majority of people who have been hospitalized in intensive care units and is the age group with the highest mortality rate (Geiss, 2020; National Epidemiological Surveillance Network, 2020). Likewise, being infected with COVID-19 increases mortality by 20% in people aged 60–69, by up to 40% in people aged 70–79, and by up to 75% in people over 80 (Barreiro and Rodríguez, 2020).

Moreover, research in the field of social representations (Smith and Joffe, 2012) and EID highlights the role that the emotional context plays in symbolic thought and its relevance in making a topic recognizable and understandable (Höijer, 2010). In SRT, the important role played by emotions is explained by emotional anchoring and emotional objectification processes (Joffe, 2002; Höijer, 2011). On the one hand, through emotional anchoring, new phenomena can be linked with emotions that are already familiar, making the unknown become known (Höijer, 2011). Many psychological research studies have demonstrated that emotions can help us to judge and interpret society-level situations and objects (Bless et al., 2004) and previous research on SRT indicates that this is also true for EID.

In fact, the work carried out so far has revealed that in modern societies there are recurring emotional patterns that emerge in response to the threat of EIDs. The most common emotion linked to EID representations is clearly the fear that is evoked not only by the threat of the disease but also by uncertainty or the unknown (Joffe, 2011; Idoiaga et al., 2017a,b). Emotions of anger are also clearly visible and are particularly evident in relation to blaming processes (Idoiaga et al., 2017a,b). Moreover, the combination of these emotions usually results in “EID fatigue” (Joffe, 2011), that is, an emotional fatigue that is a consequence of having been bombarded with a litany of imminent infectious diseases or health disasters (Joffe, 2011; Sherlaw and Raude, 2013).

In the case of research conducted with the elderly, previous studies suggest that they represent the risk posed by EIDs in emotional terms, intrinsically linking the threat to the emotions of restlessness, fear, anxiety, tension, nervousness, and disgust (Idoiaga et al., 2016). Moreover, the WHO has also warned that the risk posed by COVID-19 could generate greater distress, anxiety, anger, stress, agitation, and withdrawal in the elderly during the outbreak, or at least during the lockdown period (Wang et al., 2020; World Health Organization, 2020). In fact, studies focused on elderly people in China in the face of the COVID-19 situation show that elderly people are primarily affected in psychological and emotional terms (Meng et al., 2020) since they feel the risk of mortality linked to the age factor, which leads to the emergence of negative emotions (Qiu et al., 2020).

In addition, older adults are highly susceptible to the effects of isolation during the lockdown, which, in turn, may also have an impact on their emotional state. In fact, in recent years there has been a significant increase in the number of single-person households headed by older people (Abellán and Pujol, 2016). Therefore, social distancing can increase unwanted feelings of loneliness or solitude, exacerbating the health problems suffered by older people in the long-term (Pinazo and Bellegarde, 2018).

Further, the process of emotional objectification has a considerable emotional component. That is to say, when an EID appears, specific, frightening images are shown repeatedly (Höijer, 2010, 2011). For instance, the media shows photographs of corpses, infected people, and of scientists dressed like astronauts. Mass media makes a particular use of these images in its coverage of new events, and, consequently, emotional objectification turns several media images into icons for more abstract events (Höijer, 2010; Smith and Joffe, 2012).

Given these considerations, it is of critical importance to identify how this risk population is living through the pandemic and specifically how they deal with it at critical moments. In fact, to develop the present research a key moment was chosen – the explosion of the COVID-19 outbreak in Spain. This was the moment when the pandemic was no longer regarded as something localized that affects other countries to something that fully affects people’s own society. This particular moment was chosen because a deep understanding of how the elderly represented the pandemic during that early period may be critical to understanding the course of events. Moreover, this key moment has not been specifically analyzed in previous research.

Thus, the main goal of this study is to explore the impact of the COVID-19 outbreak on the elderly from a psychosocial perspective, with the specific aim of examining how they represented and emotionally coped during this early stage of the pandemic. Beyond this general objective, as research questions, we also intend to analyze if these representations were transformed because of the declaration of the state of alarm and lockdown. Similarly, this study also analyses if these representations and emotional patterns are consistent with those that emerged in previous EIDs. These findings are expected to be helpful in informing the development of strategies and tools that, by taking into account the needs and concerns of the elderly, will ultimately help them to overcome these extraordinary circumstances.

## Design and Method

### Sample

A total of 115 people participated in this study. The sample was recruited from the Basque Country region located in Northern Spain. Of the sample, 66% were women and 34% were men. The mean age of the participants was 67.48 years (SD = 4.70) with an age range of 60–85 years.

### Procedure

In order to access the elderly during pre-lockdown and lockdown, the associations of elderly people in the territory of the Basque Autonomous Community were asked to disseminate this research proposal (online) among their users and relatives. This was also published in the local press (newspapers, magazines, radio, and television programs) encouraging older people to participate. The questionnaires were completed from the 11th to the 18th of March 2020, with 49% of the participants completing the questionnaire before the lockdown was ordered, and the remaining 51% completing this after the lockdown period had begun. This research has obtained the approval of the Ethics Committee of the UPV/EHU [M10/2020/055].

### Data Collection Method

To analyze the participants’ social representations of COVID-19, the Grid Elaboration Method for the free association was employed, which has been useful for conducting research on social representations of global climate change, EIDs, and other issues (Joffe and Elsey, 2014; Idoiaga et al., 2017a). This method consists of providing participants with a paper with instructions and four boxes. In the instructions used here, participants were asked to write down or draw any idea that comes to their mind when they think about the word “COVID-19.” They were also asked to fill in the boxes following the order in which the contents come to their mind (i.e., to write the first thought in the first box; the second thought in the second box, etc.). All participants were asked to fill in all four boxes. Subsequently, the participants were asked to complete their response by clarifying the meaning of each of their ideas in an attempt to gather further information and explanations about the elicited items. This allowed us to obtain a complete explanation about each word or idea, which formed the basis of the subsequent analysis.

### Data Analysis Method

The Reinert method using Iramuteq software for lexical analysis (Reinert, 1983, 1990) was employed to analyze the corpus of text. This method has frequently been used for the study of social representations (Lahlou, 2001; Klein and Licata, 2003; Kalampalikis, 2005), confirming that the results obtained agree with those of other methods used in this field of research (Lahlou, 1996).

This method is based on the premise that words are not independent of each other, but reflect underlying themes. Reinert’s (1983, 1996, 2003) main thesis is that all discourse is expressed from a set of words that constitute units of meaning independently of their syntactic construction. These units of meaning evoke a way of thinking about the object being spoken of, or a field of thought, since it is from these that the statements acquire meaning.

The redundancy of successions of words, or the concatenation of words that make up a given discourse, makes it possible to locate the “lexical worlds” evoked by the enunciators (Molina-Neira, 2017). Iramuteq is a software that eliminates problems of reliability and validity in text analysis by using the Reinert method (Reinert, 1996; Klein and Licata, 2003). Specifically, the software creates a dictionary of “whole words” (nouns, verbs, adjectives, and adverbs). The initial text corpus is then broken down into segments that have the approximate length of a sentence or two (40 words; Kronberger and Wagner, 2000). The corpus is analyzed in terms of the presence of whole words in the segments. The segments and reduced forms are used to create a contingency table, which shows the distribution of vocabulary per segment. From this contingency table, the program generates a squared distance matrix, indicating that two segments are close if they share some of the words analyzed (Reinert, 1996).

Subsequently, the software, following the Reinert method, runs a descending hierarchical cluster analysis on this distance table, which yields classes of segments that best differentiate the vocabulary. In so doing, this software assists in the interpretation of texts. It extracts sets of words that are referred to as classes, which co-occur and are best differentiated from other classes. Specifically, the software identifies the words and text segments with the highest chi-square values, that is, those words and text segments that best identify each class or idea that the participants have repeatedly mentioned.

In accord with previous research using the Reinert method (Camargo and Bousfield, 2009), the raw data were entered into the Iramuteq software and the most significant items of vocabulary in each class were selected on the basis of the following three criteria: (1) an expected value of the word greater than 3; (2) proof of the chi-square association, tested against the class [*χ*^2^ ≥ 3.89 (*p* = 0.05); *df* = 1]; and (3) the word appears mainly in that class, with a frequency of 50% or more. The Iramuteq software also determines which text segments are associated with each class or group of words and classifies them according to their chi-square. In this study, the text segments with the most significant chi-squares of each class were recorded.

Once these “lexical universes” have been identified, they are associated with “passive” variables (independent variables). In the present case, the passive variable was the period during which the questionnaire had been completed, that is, before or after the state of emergency and lockdown had been declared.

Reinert method operations are statistical, transparent, and reproducible until the final stage of interpretation, where the analyst assigns a label to each specific vocabulary set that the software had identified as a lexical world on the basis of co-occurrences and distribution patterns (Schonhardt-Bailey, 2013). In the final phase, in order to create the labels or titles of each class, a systematic process was used in which two of the researchers independently named each class based on the words and associated tweets. A third researcher created a final label that was approved by all three researchers.

Finally, as a complementary analysis, Iramuteq also conducts a lexical similarity analysis. This analysis looks at the corpus in a completely different way. The approach is based on the connecting properties of the whole corpus, without taking into account the Specific Context Units or the subjects. This type of analysis considers that the more subjects treat two elements in the same way, the closer they will be (in terms of representational structure) to the object they refer to (Molina-Neira, 2017). To do this, co-occurrences between words are identified according to their connections in the text, helping to identify the structure of the text corpus contents due to its visualization in graphic form, which illustrates the content of the social representation of the object studied and its internal organization, including its common components and specificities (Marchand and Ratinaud, 2012).

Therefore, the analysis allows for defining the identity of the subjects’ representational cores, since the program identifies a semantic nucleus detected by word co-occurrences (Camargo and Justo, 2013). That is, the analysis of similarity presents a summary of the structure contained in a representation, from a tree-shaped graph that represents the maximum forms and those that are related, where the nodes are the forms and the lexical communities are shown (Ormeño, 2016), making visible “the classes constituted and the intensity of the links between the elements that make up a representation on an object” (Latorre, 2005).

## Results

**Figure 1:**
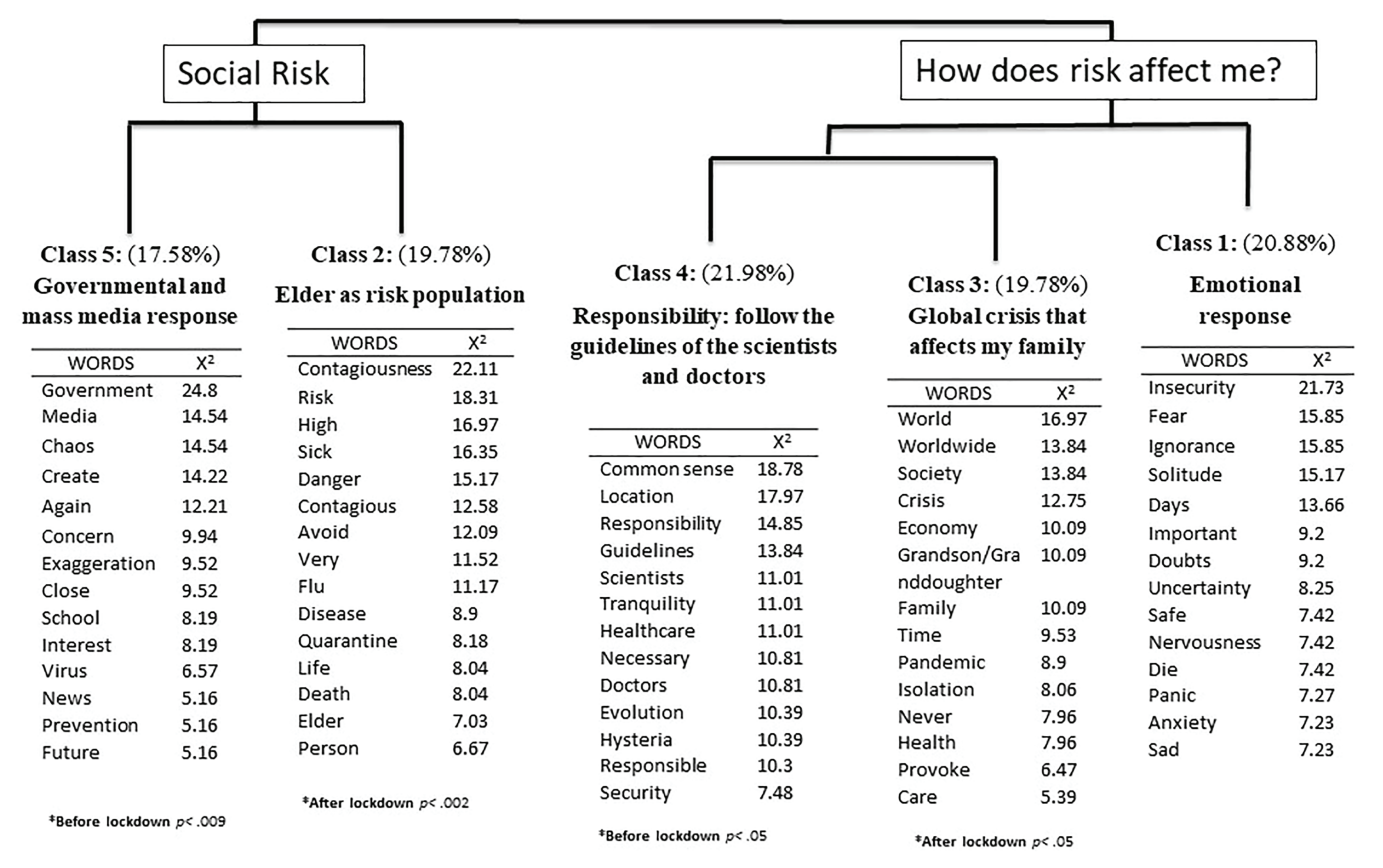
The hierarchical clustering dendrogram of the free association exercise, with the most frequent words and the words with the greatest association *χ*^2^(1), *p* < 0.001, and the associated response period (*).

First, to analyze the main representations expressed by the participants, the text corpus was analyzed using the Reinert method. This allowed for clarifying which terms were used by the elderly to represent Covid-19 and how this representation was transformed when the state of alarm and lockdown were decreed. The full corpus contained 9,004 words, of which 1,995 were unique words. Specifically, the descending hierarchical analysis divided the corpus into 221 segments and 5 classes. The results of this analysis can be observed in Figure 1.

The analysis identified the main ideas held by the participants regarding COVID-19, elicited through the free association procedure. Each issue or idea is represented by a set of typical words and text segments, which is referred to as a class. First, the results revealed two main branches or themes (composed of different classes), which are referred to as main clusters, and labeled as “social risk” and “How does the risk affect me?” The first main cluster is composed of Class 5 (government and mass media response) and Class 2 (elderly as a risk population). The second main cluster is composed of Class 3 (global crisis that affects my family), Class 4 (responsibility: follow the guidelines of scientists and doctors), and Class 1 (emotional response). Next, each of these classes will be explained in more depth.

Following the hierarchical clustering dendrogram, within the first main cluster concerning social risk, the first class to emerge was Class 5, with a weight of 17.58%, which has been labeled as “governmental and mass media response” because it describes how in those early days when the pandemic was no longer something distant that only affected China and began to fully affect Spain, there was a great feeling of chaos. Moreover, and as occurred in previous pandemics, the media were blamed for sensationalizing the news and disinformation and the government were criticized for not taking clear measures to deal with the imminent risk. In fact, the most significant words of this class are government, mass media, create, chaos, and exaggeration. Moreover, the most characteristic text segments of this class are the following: “The government’s messages are scandalous and contradictory. Being a serious public health issue, these contradictions make it difficult to understand what is happening. The media, although not all of them, but most of the television channels, broadcast images of almost dead people in the ICU and sell this issue as an alarmist show” (*X*^2^ = 101.94, woman, 70 years); “chaos, apocalypse, disinformation, absurd attitude of society towards the disease. It seems that the apocalypse is coming. Lack of information, contradiction of the government, and manipulation by the media” (*X*^2^ = 75.85, man, 65 years). This class was significantly linked to the responses given before the state of alarm and lockdown were declared (*p* < 0.009).

Within the same “social risk” main cluster, the second class emerges, labeled as “elderly as a risk population” with a weight of 19.78%. This class describes the pandemic as a real risk that can directly affect the elderly and, therefore, they are already beginning to self-catalyze as a risk group and can see that this pandemic may affect them more than the rest, so it will be essential that they protect themselves. However, as seen in the second typical segment of this class (and repeated in other segments), some of the older people talk about the pandemic targeting “the elderly” without including themselves in that group. That is, they attribute the risk to old people who are older than themselves. In this class, words such as risk, high, danger, contagious, old or person emerge and the most significant text discourses are: “I begin to have a certain restlessness, to pay more attention to what I am doing, touching, etc. and sometimes I am uncertain as to whether I have touched something, if I have washed my hands, if I should stay at home all the time. I belong to the population at risk because of my age and because I have other pathologies, which means a high probability of death” (*X*^2^ = 150.15, woman, 75 years); “The elderly are at great risk. We must therefore do everything we can to prevent them from being infected. The death of the sick people is a tragedy for the families, and for them, we must not become infected, and obviously also, to save our lives” (*X*^2^ = 115.88, man, 63 years). This class was significantly linked to the responses given after the state of alarm and lockdown had been decreed (*p* < 0.002). Therefore, it is seen that social risk flows from being mostly concentrated in the media or the government to becoming something that will pose an imminent risk to older people, even though many participants may be releasing themselves from that group.

In the second main cluster, it is evident how risk directly affects self-related issues concerning the participants and starts with the fourth class, labeled as “responsibility: follow the guidelines of the scientists and doctors” (21.98%). In this class, it is emphasized that to get out of this crisis, both on a personal level and as a community, the most important thing is prevention and to trust in the healthcare system of the country. The participants highlight the importance of acting with common sense and responsibility, as can be seen in the characteristic text segments: “We must listen to the doctors and health professionals and do everything they tell us, follow all their recommendations step by step, they are the ones who know how to control the COVID-19. We must take all the precautions that the doctors and scientists consider necessary without questioning anything, we can trust them!” (*X*^2^ = 142.77, woman, 77 years); “I will continue to use my common sense, it has worked well for me in other circumstances and I hope now it will do so too. We must trust the information given by the health professionals, even if we do not understand it very well. We have to be responsible to stop this!” (*X*^2^ = 140.14, man, 80 years). This class was significantly linked to the responses given before the state of alarm and lockdown were decreed (*p* < 0.05).

Within the same main cluster, the third class (19.78%) emerged, which has been labeled as a “global crisis that affects my family.” This class is divided into two sub-themes. On the one hand, the participants understand COVID-19 as a global pandemic, which affects the whole world not only on a health level but also on economic and social levels. This idea is represented by typical words such as world, worldwide, society, economy, or the following text segments: “This is a global crisis, but not only a health crisis, an economic crisis and a social crisis too. Let us see if from this we can learn that society demands social improvements and that regardless of where you were born, you have to face the pandemic” (*X*^2^ = 69.32, man, 72 years); “This crisis is global and we in Europe are not bad at all. Maybe I’m dying, because I’m an old man, but we’ll get through this. But what happens in Africa? Or in the United States, without public health and social services?” (*X*^2^ = 67.64, man, 81 years). But, on the other hand, the participants also make an explicit reference to a specific (and the most intimate) sphere, which is that the disease can also affect their own families, with words like grandson or granddaughter, family or care, and text segments such as: “The first thing I think about is that I have three children and six grandchildren, and I am afraid that something will happen to them. Because the virus is a threat. Up to now we have lived very peacefully and thank goodness we have a good and organized health system. That’s what gives me some peace of mind in this, it’s what’s going to solve this” (*X*^2^ = 74.74, woman, 76 years); “I am afraid for my friends and especially for my family. My grandchildren are very small, I hope nothing happens to them! We have to take care of ourselves and the people close to us” (*X*^2^ = 69.32, woman, 71 years). This class was significantly linked to the responses given after the state of alarm and lockdown had been decreed (*p* < 0.05). Therefore, it can also be observed that by declaring the state of alarm, the representations were transformed from relying mostly on prevention to focusing on how to protect the family and loved ones in the face of a global and dangerous pandemic.

Finally, and also with regard to how the risk affects the self, the first-class emerged, which concerns the “emotional response” (20.88%) experienced by the participants. This class is not significantly linked to either the pre‐ or post-lockdown period. Therefore, it was expressed in an equivalent manner throughout the response period. In this class, feelings of insecurity, fear, solitude, uncertainty, nervousness, and anxiety are stated as a response to the current health crisis. The following are some of the most significant text segments: “Fear of contagion and the risk to my life. I live alone, the fear of being sick and alone is overwhelming. Being admitted to a hospital and being alone, and above all the fear of dying alone. I cannot get these ideas out of my head. I put my attention onto other things, I try to be as positive as I can and I talk to friends to make plans for when the quarantine is over, but the fear lies within me” (*X*^2^ = 103.39, woman, 70 years); “Fear, nervousness, restlessness, and uncertainty. I feel afraid because I do not feel that the pandemic situation can be controlled, I feel helpless. I feel nervous because to solve this, many factors must be involved and that’s really difficult. Not knowing what is going to happen makes me feel insecure, in these circumstances, I am unable to focus” (*X*^2^ = 93.32, woman, 75 years); “Uncertainty, fear, doubt, and mistrust. This lack of information is what makes me feel bad emotionally. I feel very alone, even though I am with my family” (*X*^2^ = 84.18, man, 78 years).

**Figure 2:**
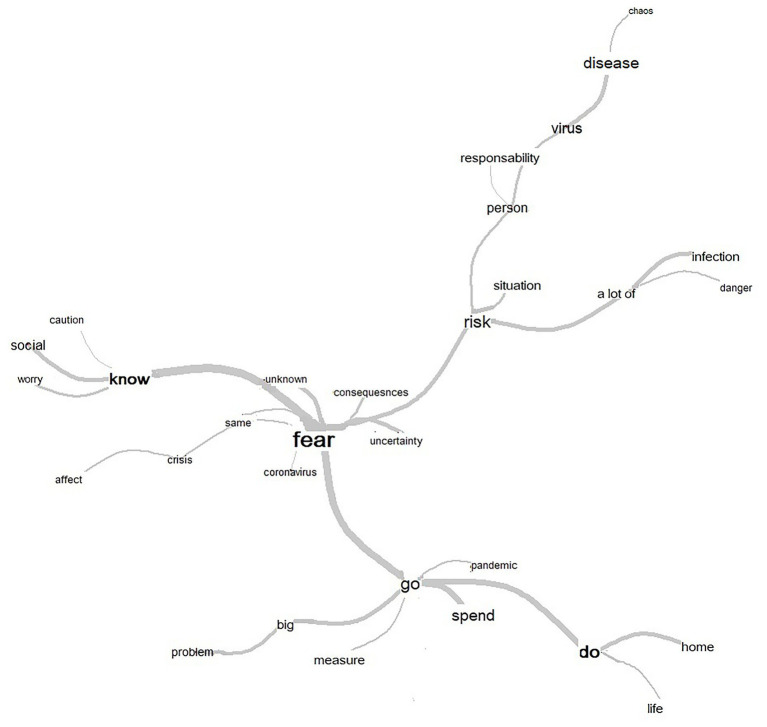
Results of the lexical similarity analysis produced by the free association exercise.

Secondly, in order to generate an image that would reflect the co-occurrences between all the words in the corpus beyond their division into classes, a lexical similarity analysis was conducted with all the words with a frequency greater than 14, the results of which are displayed in Figure 2. The idea was to analyze how the words of the corpus were interconnected on a common plane and to identify the cores or nucleus of the representations.

By default, the words are in the nodes of the graph and the edges/links represent the co-occurrence between them. The higher the frequency of the words, the greater the size of the words on the graph. The higher the co-occurrence between words, the thicker the line between them. The results of this analysis revealed that fear (*n* = 80) was the word with the highest frequency and the core or nucleus of the emerged representation. Fear was represented as being linked to uncertainty (*n* = 19) and emerged because the unknown (*n* = 14) consequences (*n* = 16) of the COVID-19 (*n* = 14) and because this crisis (*n* = 16) is going to affect (*n* = 14) them. Indeed, fear was also linked to risk (*n* = 28). Risk is assumed because a lot of (*n* = 23) infections (*n* = 20) are occurring and old people feel that they are in danger (*n* = 17). To face this risk, people must be responsible (*n* = 18) because if not, the virus (*n* = 34) and the disease (*n* = 35) will create a situation of chaos (*n* = 14). Likewise, fear was linked to the new pandemic (*n* = 23) of which society has to go on (*n* = 50), this is expected to be a big (*n* = 22) problem (*n* = 21) and therefore strict measures (*n* = 17) are going to be applied, this is why now time should be spent doing things (*n* = 49) at home (*n* = 20), and in life (*n* = 18). Finally, old people feel fear because they know (*n* = 36) this is a social issue to be addressed with caution (*n* = 20) and worry (*n* = 19).

Thus, fear was viewed as a central emotion in the social construction of COVID-19 and it appears that it belonged to the world of unfamiliarity and the threat of the unknown. Fear was related to risk regarding the contagious capacity of the disease and the danger perceived by the population that felt most threatened by COVID-19. It was also related to the high impact that it had on the lifestyle of the elderly, who were forced to manage it in their everyday lives.

## Discussion and Implications

This research has provided important clues for identifying how older people integrate the COVID-19 crisis into their everyday thinking. Our findings have revealed two main issues that are aroused in the consciousness of the elderly. These issues are related to (a) social risk, and (b) how the risk affects them directly. Moreover, interesting results have also been found regarding the way in which elderly people cope with the pandemic at an emotional level.

In the main cluster labeled as “social risk,” the voices of the elderly reveal the ways in which they represent a number of issues concerning social risk management. In the “governmental and mass media response,” they criticize the stance adopted by both the government and the mass media (among other issues) on the grounds that they (the government and mass media) do not convey a clear message and that the information given is both insufficient and contradictory. Representing the government as an agent to be considered in the management of the crisis is a novel aspect that has emerged in other investigations of COVID-19 representations in the general population, in which the issue of government was not even mentioned (Idoiaga et al., 2021b). Nonetheless, in other previous EID studies, the role of the government and media has been disputed, whilst they have also been accused of using fear for their own interests (Washer, 2010; Wagner-Egger et al., 2011; Idoiaga et al., 2017b).

A further issue that was mentioned with respect to the management of the crisis was the impact of the situation on the elderly as a risk population. Words such as risk, danger, or contagious appear, all of which are related to the status of being elderly. This representation is logical since in this pandemic it has been stressed from the outset that older people are the largest global risk group (World Health Organization, 2020). However, this point can also be contradictory, because sometimes it might be difficult to pinpoint precisely who is part of this “elderly risk group.” For instance, the Spanish government published specific recommendations for older people, advising them to remain in complete lockdown, to take hygiene measures, and to call family and friends on a daily basis (Spanish Ministry of Health, Consumers and Social Welfare, 2020). However, one problem is that whilst there are higher mortality rates from the age of 60 onwards, the mass media tend to place special emphasis on those aged over 80 (BBC, 2020). Therefore, deciding exactly who belongs to the risk group and whether or not they themselves belong to it can be a source of confusion for many people aged over 60. Moreover, this could create a high level of risk, since wanting to protect one’s identity from vulnerability can lead many people to associate the risk with people older than themselves (Idoiaga et al., 2016) creating othering processes (Joffe, 2011). In fact, it should be noted that this class appears in the results far from how risk affects oneself, which is represented in the other main cluster.

The second main cluster, which is labeled as “How does risk affect me?”, describes a number of issues that are more strongly linked to the notion of the *self* in elderly people. Firstly, our elderly participants point out the necessity or responsibility for following the advice of scientists and doctors. In this crisis, it seems to them that health workers are the heroes or the reliable sources to be followed, and that they must comply with everything that they are asked to do by such figures (Wagner-Egger et al., 2011). It is striking how, in terms of the figures that they follow for advice, it is the scientists and doctors who are represented as key points of reference as opposed to politicians and government. This is particularly interesting, given that since the state of emergency was declared, the prime minister has taken almost complete control of the country (Presidency of the Government of Spain, 2020). This is most likely due to the lack of confidence in the government for the way that they have managed EIDs and the lack of clarity in their messaging whilst addressing this crisis, as already pointed out elsewhere (Washer, 2010; Idoiaga et al., 2017b).

Furthermore, whilst old people acknowledge that this pandemic is something that affects the entire world, they also show concerns for their family, giving particular mention to their worries regarding their grandchildren. Hence, in this outbreak it is also evident that there is a globalization of risk, affecting both worldwide and personal spheres (Beck, 2009). Moreover, participants are also worried because this crisis may have consequences not only at a health level but also at social and economic levels, and they are particularly afraid of the economic crisis that this situation might inevitably bring.

Emotional response also emerged in the social representations of the elderly. First, it should be noted that, as depicted in the similarity analysis (Figure 2) the nucleus or core of the representation that old people hold about the pandemic is articulated around fear, in a clear pattern of emotional anchoring (Höijer, 2011). This emotional pattern is somewhat recurrent during the first phase of health epidemics (Idoiaga et al., 2016) and usually casts society into an emotional whirlwind (Strong, 1990). That is, through fear-related emotions the new risk of COVID-19 is understood and incorporated into a familiar representation (Höijer, 2011). Therefore, it would be interesting to analyze, as several researchers have suggested (Höijer, 2011), how emotional anchoring could make many phenomena comparable. That is, how some emotions are the core or the nucleus of social representations of life-issues (Wagner et al., 1996) such as fear in the case of EID, climate change, environmental risks, or terrorism.

Our results, however, go beyond that emotional pattern. In fact, we have been able to see that when people are asked freely – in free association and without any reference being made to emotions – they mention insecurity, solitude, uncertainly, nervousness, and anxiety, a wide range of emotions hidden or rooted behind that “fear.” Thus, all those emotions might also be part of the emotional anchoring process. Some of these feelings are also recurrent since they have been identified in previous research about EIDs and the elderly, along with emotions of restlessness, fright, tension, and disgust (Idoiaga et al., 2016). Nevertheless, the feelings of solitude and loneliness are new. Whilst to some extent these feelings could be linked to the confinement that has been imposed by the lockdown measures, it should be noted that these emotions have not been reported in similar research studies of COVID-19 in either young people (Idoiaga et al., 2021a) or the general population (Idoiaga et al., 2021b). According to Berger and Poirie (1995), loneliness is an exceedingly painful experience that is the sum of an unfulfilled need for intimacy and social relationships that are felt to be insufficient or not entirely satisfactory. Therefore, the findings reported here are worrying, since social isolation in the aging population has been shown to have profound negative effects on longevity and physical and mental health (Olsen et al., 1991), creating problems such as sleep disturbances, depression, and fatigue (Choi et al., 2015).

Second, emotional objectification turns particular images into icons for more abstract events (Smith and Joffe, 2009; Höijer, 2010). One of the recurring concerns or complaints of participants in this study is that the mass media repeatedly show specific, frightening images linked to COVID-19 (Höijer, 2010, 2011). This media coverage was represented with a clear link to the blaming processes and to a highly emphasized emotional charge of anger. Therefore, it should be analyzed whether this process of emotional objectification is built on anger and what possible consequences this pattern might have.

This research work has therefore confirmed that when social representations were formed in order to understand the emergence of the COVID-19 crisis, the emotional response conditioned this understanding of the risk. So, in a complete response to the pandemic, the importance of taking into account emotional anchoring and objectification is unquestionable because these will influence the distribution of the size, form, and time of the political and social response to the crisis.

Finally, it is also worth remembering that social representations are transformative processes that are in constant motion (Moscovici, 1998; Joffe, 2003). In fact, our results indicate that there was a body of cognitive transformation – rather than emotional – during the week of analysis. Therefore, although the analysis of this first phase, where the pandemic went from something distant to something that completely influenced the lives of the participants, is of special interest, it would also be very interesting to analyze how the representations of COVID-19 are transformed along with their emotional patterns throughout the crisis.

To conclude, it is also important to acknowledge the limitations of this research. To begin with, this study worked with a non-probabilistic sample and employed a cross-sectional design, whilst also located in a specific context – the North of Spain. Therefore, any conclusions cannot be generalized to any society or context. Further, we should also consider the online format used to implement the research. This format may have created bias, particularly when it comes to reaching older or less connected participants, but, due to the pandemic, social distancing was crucial, and this was the most practical way of carrying out the study.

In short, we are experiencing an unprecedented and rapidly changing situation. Understanding the patterns of thinking linked to the current pandemic from the voice of the more vulnerable members of society, the elderly, is of vital importance. In particular, identifying how they cognitively represent and how they emotionally face (by anchoring and objectification processes) this new situation provides us with valuable information for identifying the strategies they can use to cope with the problem from a psychological and social perspective. As a starting point, the findings of this research make it clear that when referring to elderly people as a risk group, there is a need to specify precisely the age group to which this term refers and propose specific recommendations for each case. In other words, it is vitally important to be as direct and clear as possible. Moreover, special attention must be paid to the central importance of fear and the emergence of feelings of solitude. In this regard, it is critical that the government and local authorities develop social and inclusive policies to help the elderly alleviate the potential effects of confinement by addressing their psychological, social, health, and well-being needs.

## Data Availability Statement

The raw data supporting the conclusions of this article will be made available by the authors, without undue reservation.

## Ethics Statement

The studies involving human participants were reviewed and approved by Ethics Committee of the UPV/EHU [M10/2020/055]. The patients/participants provided their written informed consent to participate in this study.

## Author Contributions

All authors listed have made a substantial, direct and intellectual contribution to the work, and approved it for publication.

### Conflict of Interest

The authors declare that the research was conducted in the absence of any commercial or financial relationships that could be construed as a potential conflict of interest.
